# The Laws of Health

**Published:** 1884-10

**Authors:** 


**The Laws of Health.** Physiology, Hygiene, Stimulants and Narcotics. For Educational Institutions and General Readers. Copiously illustrated. By Joseph C. Hutchinson, M. D., LL. D., Author of a Treatise on Physiology and Hygiene; late President of the Medical Society of the State of New York. Clark & Maynard, 734 Broadway, New York.

This little book of about 200 pages presents, in clear and concise language, the knowledge of to-day concerning the laws of health and the effects of narcotics and stimulants, as far as can be expected or desired in a work so elementary. It is specially designed to meet the requirements of grammar schools and academies. It is one of the best school manuals, and may well receive the commendation of the physician.

